# Uncommon Large and Bilateral Fibrous Cephalic Plaques in a Patient with *TSC2*-Related Tuberous Sclerosis Complex

**DOI:** 10.3390/children10101614

**Published:** 2023-09-28

**Authors:** Ariadna González-del Angel, Adriana Ruiz-Herrera, Nancy Leticia Hernández-Martínez, Carlos G. Todd-Quiñones, Carola Durán-McKinster, Patricia Herrera-Mora, Miguel Angel Alcántara-Ortigoza

**Affiliations:** 1 Laboratorio de Biología Molecular, Subdirección de Investigación Médica, Instituto Nacional de Pediatría, Ciudad de México 04530, Mexico; ariadnagonzalezdelangel@gmail.com (A.G.-d.A.); yazzama@gmail.com (N.L.H.-M.); 2 Servicio de Genética, Hospital Médica Campestre, León 37180, Guanajuato, Mexico; ruizhadriana@hotmail.com; 3 Licenciatura en Enseñanza y Aprendizaje en Telesecundaria, Benemérita Escuela Normal Veracruzana “Enrique C. Rébsamen”, Xalapa 91017, Mexico; cgtoddq@gmail.com; 4 Departamento de Dermatología, Instituto Nacional de Pediatría, Ciudad de México 04530, Mexico; caroladmc53@gmail.com; 5 Departamento de Neurología, Instituto Nacional de Pediatría, Ciudad de México 04530, Mexico; pherreramora@prodigy.net.mx

**Keywords:** fibrous cephalic plaque, atypical fibrous cephalic plaque, bone hyperostosis, calvaria hyperostosis, cutaneous marker, tuberous sclerosis complex, *TSC2*

## Abstract

Tuberous sclerosis complex (TSC) is a genetic disorder, frequently characterized by early dermatological manifestations. The recognition and adequate description of these dermatological manifestations are of utmost importance for early diagnosis, allowing for the implementation of therapeutic and preventive measures. Fibrous cephalic plaques (FCPs) are considered a major diagnostic criterion for TSC, as FCPs are the most specific skin lesions of TSC. The localization, consistency, color, and size of FCPs vary widely, which can cause diagnostic delay, especially in patients with atypical presentations. The present report describes a female TSC patient with a confirmed heterozygous pathogenic genotype, NG_005895.1 (*TSC2*_v001): c.2640-1G>T, who presented with uncommon large and bilateral FCPs causing bilateral ptosis and marked with hyperostosis of the diploe that generated an asymmetry of the brain parenchyma. Differential diagnoses considered initially in this patient due to the atypical FCPs are described.

## 1. Introduction

Forehead plaques or fibrous cephalic plaques (FCPs) are skin lesions observed in 19–46% of patients with tuberous sclerosis complex (TSC, MIM #613254, #191100), and are considered a major diagnostic criterion of this disease [1,2]. Although these lesions usually appear unilaterally on the forehead, they may be observed in other parts of the face or scalp [1]. Clinically, they are irregular hamartomas that can vary in consistency, color (ranging from skin-colored to yellow or brown), and number [1,3]. FCPs arise as plaques rather than papule confluences; moreover, plaque size may vary, reaching up to several centimeters in diameter [1,3,4].

TSC is caused by loss-of-function genetic variants in the tumor suppressor genes *TSC1* (MIM #191100) and *TSC2* (MIM #613254). Some of the dermatologic manifestations of TSC, including FCP, have been reported to be more frequent in patients with pathogenic variants of *TSC2* than of *TSC1* (40% vs. 10%) [5]. 

To the best of our knowledge, no previous clinical report has described large, bulky FCPs causing bilateral ptosis associated with an atypical hyperostosis of the diploe that conditioned an asymmetry of brain parenchyma in a patient with molecularly confirmed TSC.

## 2. Case Report

**Figure 1:**
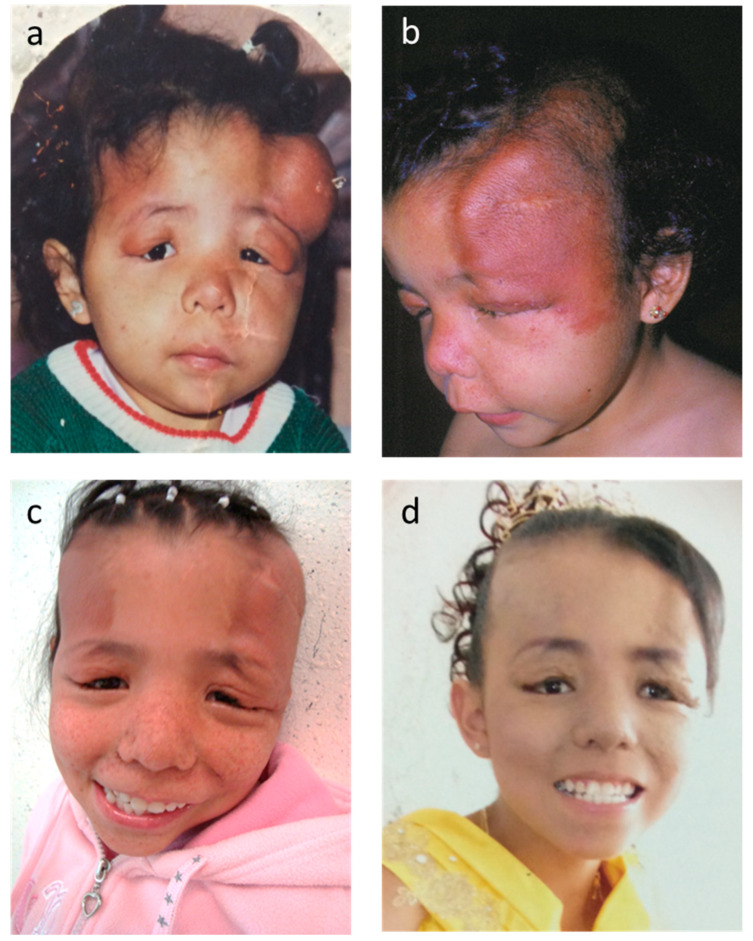
Images of the face of the present patient. (**a**) Large and bulky plaques causing palpebral ptosis. (**b**) Images at an age of 16 months, showing large alopecic brownish-red-colored plaques with smooth surfaces. (**c**) Image at 10 years of age after palpebral and forehead surgery to remove FCPs and the use of skin tissue expanders to correct alopecia; numerous facial angiofibromas were also present. (**d**) Image at 15 years of age after removing the FCPs.

The patient was a Mexican female who was the fourth child of a young and healthy non-consanguineous couple and had three healthy older siblings. During pregnancy, macrosomia was detected, and a cesarean section was indicated. The patient was born at full term with a weight of 5240 g; the length and Apgar score are unknown. It was referred that she had bilateral and asymptomatic large and bulky dermatological plaques on the forehead (Figure 1a) since her birth. Psychomotor development was normal until the age of 8 months, when she experienced infantile spasms followed by focal seizures. At that time, she was referred to our institution.

Physical examination at 16 months of age revealed weight and height within the normal percentiles. Examination of her frontal-parieto-temporal regions and eyelids showed bilateral, well-defined alopecic firm plaques, 8 and 10 cm long each, that were brownish-red in color and had a smooth surface, causing bilateral ptosis (Figure 1b) without follicular comedo-like openings. Five oval hypopigmented macules, each approximately 1 cm in diameter, were present on her trunk. 

Subsequently, the patient also exhibited bilateral retinal hamartomas, cortical tubers, subependymal nodules, renal angiomyolipomas, and a cardiac rhabdomyoma, which regressed spontaneously. Collectively these manifestations supported a definite clinical diagnosis of TSC [2]. 

**Figure 2:**
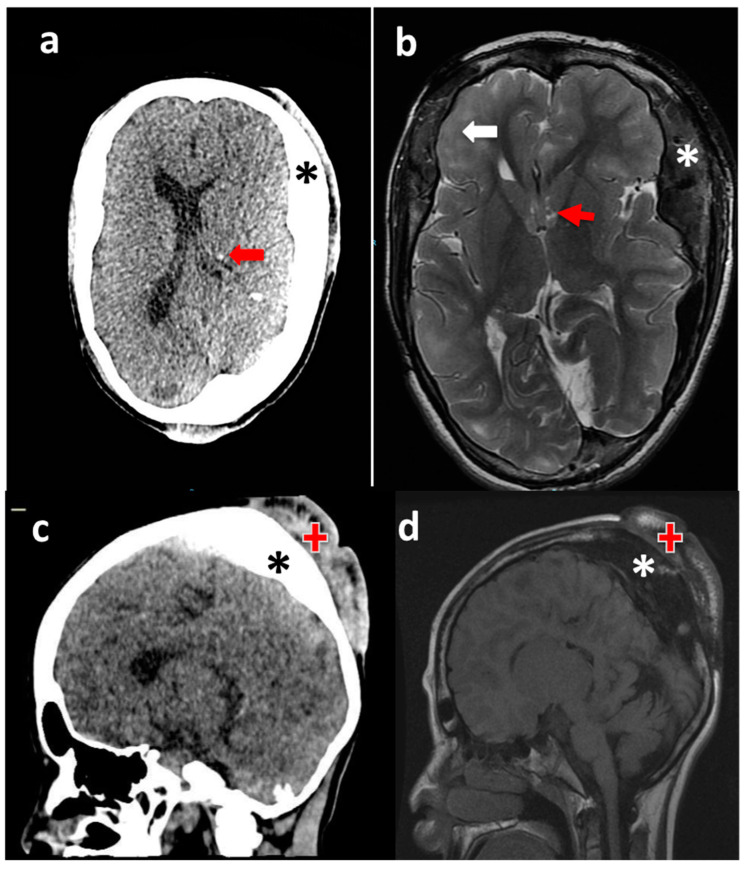
Cerebral images of the patient. (**a**) CT scan of the skull, showing enlargement of the diploe (asterisk) and calcified subependymal nodules (red arrow). (**b**) Brain MRI showing an atypical hyperostosis of the diploe (asterisk) with asymmetry of the brain parenchyma, cortical tubers (white arrow) and subependymal nodules (red arrow). (**c**) Comparison of CT and (**d**) MRI showing hyperostosis of the diploe (asterisks) and the lack of connection of the FCP (red crosses) with the calvaria and brain.

A computed tomography (CT) scan of the patient’s skull showed enlargement of the diploe and calcified subependymal nodules (Figure 2a). Brain magnetic resonance imaging (MRI) scans showed an atypical enlargement of the diploe, resulting in an asymmetry of the brain parenchyma. The MRI scans also showed cortical tubers and subependymal nodules (Figure 2b). Both the CT (Figure 2c) and MRI (Figure 2d) scans showed that the FCPs did not communicate with the calvaria or brain.

The patient underwent a partial resection of the eyelids and frontal plaques at age 16 months. Skin biopsies showed normal epidermis with abundant collagen fibers forming nodules surrounding the gland ducts, hair follicles, and vessels in the deep dermis. These findings were compatible with FCP, as no follicular comedo-like openings or follicular-sebaceous cystic dilations were present [4,6]. 

At the age of 10 years, the patient underwent a resection of the FCPs and skin tissue expansion to correct alopecia (Figure 1c). At the age of 15 years (Figure 1d), the patient exhibited moderate intellectual disability, but was capable of basic self-care; her seizures were controlled with vigabatrin and she had attained a breast and pubic Tanner pubertal stage of 2, but had not yet achieved menarche. She achieved menarche at the age of 16 years, but underwent an elective hysterectomy at the age of 17 years. She was last clinically evaluated at the age of 18 years, at which time she weighed 43.8 kg (below the third percentile), was 164.6 cm in height (fiftieth percentile), and experienced refractory tonic-clonic seizures.

Both parents were normal upon physical examination, cerebral neuroimaging, ophthalmological evaluation, and renal ultrasound. 

## 3. Genetic Analyses

**Figure 3:**
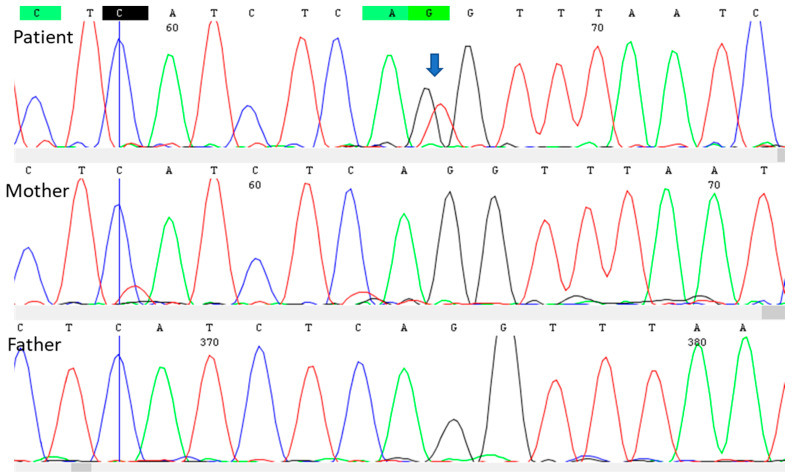
Electropherogram of the forward strand of genomic DNA from the patient and her mother and father. The arrow indicates a heterozygous NG_005895.1 (TSC2_v001): c.2640-1G>T genotype in the patient. This pathogenic variant abolished the acceptor splicing site at intron 23. The *TSC2* genotypes in both parents were normal.

DNA samples were obtained from peripheral blood leukocytes of the patient and her parents using the standard in silica adsorption method. Conventional Sanger sequencing of the patient’s DNA identified a heterozygous genotype that eliminates the splice acceptor site at intron 23 of the *TSC2* gene (NG_005895.1 (TSC2_v001): c.2640-1G>T; LOVD TSC2_004263 Variant #0000598059). This variant was previously described by our group [7]. This pathogenic variant was not identified in the DNA of either parent, suggesting that this mutation had arisen de novo in the present patient (Figure 3).

## 4. Discussion

It has been reported that 12% of patients with TSC, especially those without epilepsy, are not diagnosed until adulthood [1]. This delay in diagnosis is partially associated with poor knowledge of the health personnel to recognize skin lesions such as FCPs, despite skin manifestations occurring early in patients with TSC, being present at birth or in early childhood [1]. Moreover, skin lesions are common in patients with TSC and should lead to the suspicion of TSC. Diagnosis in patients with an uncommon clinical presentation, however, might require other types of analysis, as has occurred in the present patient.

A study of 113 adults with TSC found that 41 (36%) had FCPs; of the latter, 29 (71%) had a solitary lesion, 12 (29%) had multiple lesions, and 20 (49%) were positive for FCP at birth or during the first two years of life. Interestingly, only 8 of 62 (13%) FCPs localized to the forehead, face, neck or scalp were ≥5 cm in size [1]. That study, however, did not mention whether any patient had an FCP as large or bulky as those observed in the present patient that caused bilateral ptosis associated with an atypical hyperostosis of the diploe that conditioned an asymmetry of the brain parenchyma.

A previous case report described a 29-year-old male with a large and bulky cutaneous vascular lesion that covered the entire right side of his face [8]. Pathologic examination was inconclusive, as the number and size of sebaceous glands had increased, the epidermis was indicative of papillomatosis with invaginations into the dermis, and there were epidermal inclusion cysts and fibrosis hyalinization and collagenization in the deeper layer of the dermis. That patient was diagnosed with TSC on the basis of the presence of intellectual disability, seizures, facial angiofibromas, and shagreen patches, but this diagnosis was not corroborated by molecular analysis. Despite the cutaneous facial lesion of the patient being large in size, it did not involve the palpebral region, and images of the diploe and brain were not provided.

Due to the uncommon FCPs observed in the present patient, nevus sebaceous of Jadassohn and nevus psiloliparus were initially considered in the differential diagnoses prior to obtaining the skin biopsy report. The dermatological lesions observed in patients with nevus sebaceous are lineal or oval plaques with alopecia, color ranging from skin-colored to yellowish-orange or brownish-black, and a smooth, nipple-like or verrucous appearance [9]. Although some of these characteristics were observed in the FCPs of the present patient, nevus sebaceous is usually present as part of the linear nevus syndrome or Schimmelpenning–Feuerstein–Mims syndrome (MIM #163200), which have multiple extracutaneous manifestations that our patient did not have [9]. Nevus psiloliparus is a soft, subcutaneous mass with a demarcated area of alopecia, as well as being a common skin marker for the diagnosis of encephalocraniocutaneous lipomatosis (MIM #613001). This latter condition is also characterized by unilateral porencephalic cysts, ipsilateral lipomatous hamartomas of the scalp–eyelids–eye globe, cortical atrophy, cranial asymmetry, developmental delay, and seizures [10]. Nevus psiloliparus was considered in the differential diagnosis of the present patient due to the presence of large areas of alopecia. 

FCPs should also be differentiated from other dermatologic lesions, such as folliculocystic and collagen hamartomas (FCCHs), which have been described as exophytic plaques with an elastotic consistency and comedo-like openings, and are thought to be considered a new clinical manifestation of TSC [6]. FCPs and FCCHs share some histopathologic features, such as collagen deposits, capillary dilation, and concentric fibrosis around follicles. However, FCCHs are also characterized by dilatation of the infundibular portion of the follicle and the presence of comedo-like openings [6]. Histopathologic evaluation of the present patient indicated that the large and bulky lesions were diagnostic of FCPs.

FCPs may also be an early cutaneous marker of the central nervous system involvement in patients with TSC [11]. Consistent with this, the present patient had moderate intellectual disability and seizures.

Sclerotic bone lesions (SBLs) are the only skeletal finding included as a minor diagnostic criterion of TSC [2]. SBLs mainly affect the vertebrae, but can also be present in other areas, such as the skull and pelvis. Other skeletal abnormalities described in patients with TSC include cystic changes in the bones of the hands and feet, local periosteal thickening along the shaft of tubular bones, hyperostosis of the calvaria, and fibrous dysplasia of the skull [12]. The present patient exhibited hyperostosis of diploe, but a bone skull biopsy was not obtained for diagnostic purposes. Interestingly, in the literature, only another patient was found to have a large dermatological facial plaque adjacent to focal hyperostosis of the calvaria. In this patient, the diagnosis of TSC was established by the presence of seizures, subependymal nodules, and shagreen patches, but it was not corroborated by molecular analysis and biopsy of the bone lesion was not performed [13]. Because skeletal examination is not included in the surveillance recommendations from the International TSC Consensus Group [2], there is not sufficient clinical information about skeletal manifestations and their prognosis in TSC patients, so it would be relevant to perform more studies about these findings in the future.

Although a clear genotype–phenotype correlation has not been identified, patients with pathogenic variants in *TSC2* have been reported to have more severe phenotypes than patients with variants in *TSC1* [5,14]. Moreover, patients with de novo pathogenic variants in *TSC2* have been reported to have a more severe phenotypic spectrum than patients with familial variants in this condition [14]. The severe cutaneous phenotype documented in the present patient supports these assumptions.

FCP has been reported to be more frequent in patients with nonsense or frameshift variants in *TSC2* [14]. Examination of the literature and *TSC2* gene mutation databases identified only one corroborated genetic variant at the same splicing acceptor site as in the present patient (c.2640-1G>A. LOVD TSC2_002213, Variant #0000782382). Phenotypic information was not reported for this individual, preventing a determination of whether this type of genetic variant contributed to the severe phenotype observed in the present patient.

## 5. Conclusions

FCP is a major criterion for the diagnosis of TSC, as it is the most specific skin lesion present before the onset of other clinical manifestations [2]. Children with this dermatologic manifestation likely have TSC, even in the absence of seizures, and should be thoroughly evaluated because early TSC diagnosis may allow the application of therapeutic measures (e.g., administration of anticonvulsants) to avoid some sequelae [1,2]. Other differential diagnoses should be considered, especially if patients have uncommon FCPs, as in the present report. Further study of skeletal involvement in TSC is warranted, with a special focus on skull lesions, to improve knowledge of its pathophysiology, prognosis, and treatment.

Furthermore, molecular tests are critical for the integrated management of patients with TSC and their families. Molecular tests can confirm diagnoses and define a possible genotype–phenotype correlation, as in the present patient, who had atypical, large and bilateral FCPs with hyperostosis of diploe.

## Data Availability

The datasets used and/or analyzed during this study are available from the corresponding author upon reasonable request.
